# The Diagnostic Utility of PCR in FFPE Skin Biopsies with Inconclusive Histopathology for Suspected Cutaneous Leishmaniasis: A Pilot Study from Colombia

**DOI:** 10.3390/tropicalmed10080232

**Published:** 2025-08-18

**Authors:** Julio César Mantilla, Nathalia Andrea Bueno, Juan Pablo Alvarez, Maria Paula López, Martha Lucía Díaz

**Affiliations:** 1 Structural, Functional and Clinical Pathology Group (PAT-UIS), Pathology Department, School of Medicine, Universidad Industrial de Santander, Bucaramanga 680002, Colombia; jumantil@uis.edu.co; 2 Immunology and Molecular Epidemiology Group (GIEM), School of Microbiology, Universidad Industrial de Santander, Bucaramanga 680002, Colombia; nathaliabuenoariza@gmail.com (N.A.B.); juan2201700@correo.uis.edu.co (J.P.A.); maria.paulalv7@gmail.com (M.P.L.)

**Keywords:** cutaneous leishmaniasis, PCR, ITS1, mini-exon, FFPE, histopathology, molecular diagnosis

## Abstract

Cutaneous Leishmaniasis (CL) is a tropical disease endemic in many regions of Latin America. Its clinical diagnosis is often supported by histopathological analysis of skin biopsies; however, parasite detection by microscopy can be challenging, particularly in chronic or pauciparasitic lesions, leading to inconclusive results. Objective: This study aimed to evaluate the utility of polymerase chain reaction (PCR) as a confirmatory diagnostic tool for CL in formalin-fixed, paraffin-embedded (FFPE) biopsies with inconclusive histopathology. Methods: We analyzed 16 FFPE skin biopsy samples from patients with clinical suspicion of CL. All cases underwent routine histopathological evaluation using hematoxylin and eosin staining and were classified as inconclusive. DNA was extracted and PCR was performed, targeting the ITS1 and miniexon regions of *Leishmania* spp. Results: PCR successfully amplified *Leishmania* DNA in 8 (50%) out of 16 samples when both targets were utilized, confirming infection. These findings highlight the additional benefits of molecular tools in cases with inconclusive histopathology, thereby enhancing diagnostic accuracy and enabling species-level identification in certain instances. Conclusions: PCR proved to be a valuable diagnostic complement to histopathology in clinically and histologically suspected cases of CL without visible parasites. Its implementation may improve diagnostic accuracy and timely treatment in endemic areas.

## 1. Introduction

Cutaneous Leishmaniasis (CL) is a neglected tropical disease caused by protozoan parasites of the genus *Leishmania*, transmitted by the bite of infected sandflies [1]. It is endemic in many parts of the world, particularly in the Americas, the Mediterranean Basin, the Middle East, and Central Asia [2]. Colombia, Brazil, and Peru collectively represent over 80% of reported CL cases in the Americas. Between 2001 and 2023, the region reported an astonishing 1,178,436 cases, averaging 51,236 cases per year. This high number of cases underscores the urgent need for improved diagnostic methods to manage the disease effectively [3]. Notably, Colombia reported 1244 cases of Leishmaniasis in 2024, with 440 of these cases occurring in the department of Santander [4]. This highlights the continued public health relevance of CL in the region, where the disease remains endemic and poses persistent challenges for accurate diagnosis and effective case management.

CL is considered a significant public health issue due to its potential to cause chronic morbidity, disfiguring skin lesions, and lead to severe social stigma, especially for women and children [5,6]. CL presents a broad clinical spectrum ranging from papules and nodules to ulcerative or infiltrative lesions, which makes its diagnosis particularly challenging for healthcare professionals [7]. In endemic regions, this clinical heterogeneity often overlaps with other dermatological conditions, such as bacterial or fungal infections, vasculitis, or cutaneous malignancies, which complicates accurate recognition and delays appropriate treatment [8,9]. As a result, the diagnosis based solely on clinical features is often insufficient, especially in chronic cases [10]. In our region, the laboratory diagnosis of CL primarily relies on the microscopic identification of the parasite in skin smears or biopsy specimens. However, direct microscopy fails to detect the parasite in a significant number of cases, particularly in chronic lesions or those with low parasite burdens [11]. Histopathology may reveal granulomatous inflammation suggestive of Leishmaniasis; however, the absence of visible parasites can lead to inconclusive or non-definitive diagnoses [12]. Therefore, the accurate identification of CL requires a comprehensive diagnostic approach that integrates clinical evaluation, histopathological analysis of skin biopsies, and, when necessary, molecular confirmation [13]. Polymerase Chain Reaction (PCR) has become an increasingly important tool in endemic settings, as it enables the sensitive and specific detection of *Leishmania* DNA from clinical specimens, including formalin-fixed paraffin-embedded (FFPE) or fresh tissue biopsies [14]. PCR amplifies the DNA of the parasite, making it easier to detect even in cases with low parasite loads. FFPE skin biopsies represent a valuable resource for molecular diagnosis, as these specimens are routinely collected for histopathological analysis and can be stored for extended periods without significant DNA degradation [15]. Using FFPE samples for PCR allows retrospective studies, facilitates diagnosis in cases where fresh tissue is unavailable, and enables molecular confirmation in lesions with low parasite loads.

Furthermore, the integration of PCR with FFPE material is not just a technical advancement, but a reliable support system in the field of CL diagnosis. It enhances diagnostic yield without additional invasive procedures, making it especially advantageous in resource-limited or retrospective clinical settings. In this study, we aimed to evaluate the diagnostic utility of PCR in a pilot study using FFPE samples from patients with suspected CL and histopathological findings suggestive of the disease but without visible parasites. Our findings not only validate the role of molecular methods in supporting the diagnosis of CL but also reassure us of the method’s reliability in cases with inconclusive conventional histopathology.

## 2. Materials and Methods

### 2.1. Sample Collection

Sixteen FFPE skin biopsy specimens were retrospectively selected from patients with chronic cutaneous lesions clinically suspected to be CL. These specimens had been archived in the Pathology Department at the Industrial University of Santander over the last four years, from January 2021 to December 2024. All skin samples were obtained from patients residing in the Santander department (Colombia). The clinical suspicion of CL relied on the epidemiological context, lesion morphology, and chronicity. All biopsies had previously been evaluated by histopathology and were classified as inconclusive or suggestive, defined as the presence of inflammatory histological patterns compatible with CL (e.g., lymphohistiocytic or granulomatous infiltrates) but without demonstrable amastigotes on hematoxylin and eosin (H&E) staining. The inconclusive classification was due to the absence of amastigotes, which are the definitive diagnostic feature of CL, in the H&E-stained sections.

Two FFPE skin biopsy samples were included as positive controls. These samples had a confirmed diagnosis of CL based on the direct observation of amastigotes in Giemsa-stained smears. Two FFPE skin biopsy samples from a non-leishmaniasis case (non-ulcerative dermatological condition) were included as a negative control to assess potential non-specific amplification or contamination. The FFPE tissue blocks were processed using the same DNA extraction and PCR protocols as those applied to the study samples.

### 2.2. Histopathological Analysis

Skin biopsy samples were stained with hematoxylin and eosin (H&E) and examined using light microscopy. Histological slides were re-evaluated independently, blinded to prior pathology reports and PCR results. They were evaluated based on their inflammatory reaction patterns in the epidermis and dermis, with the inflammatory infiltrate classified as mild, moderate, or severe. The presence of plasma cells and lymphocytes, as well as the distribution pattern of the infiltrate, was recorded, along with observations of histiocytes, including the presence of multinucleated giant cells such as Langhans-type cells. The epidermal features assessed included ulceration, acanthosis, hyperkeratosis, and spongiosis. We analyzed the dermis’s granulomatous pattern, fibrosis, vascularization, and telangiectasia. Finally, the presence of amastigote forms of *Leishmania* was assessed in all samples. The presence of lymphocytes and plasma cells was assessed on the following two scales: a predominant or considerable number of cells (2–3+), or a not seen or minority of cells (0–1+).

### 2.3. DNA Extraction from FFPE Tissues

An adapted methodology based on the protocol described by Casaril et al. [16] was utilized for DNA extraction from FFPE samples. Following these steps, a micron cutter with a no. 5 scalpel blade excised 1 mm-thick film-like slices from the paraffin block. These slices were placed in 400 µL of 5% Chelex-100 solution, prepared in 10 mM Tris-HCl (pH 8.0) and 1 mM EDTA, and then sterilized by filtration. They were subsequently boiled for 10 min. Subsequently, the samples were centrifuged at 12,000 rpm for 10 min to remove the paraffin. Tissues were rehydrated with ethanol and washed with 10 mM Tris-HCl (pH 8.0) and 1 mM EDTA. Tissue digestion was performed using a lysis buffer composed of 20 µL of proteinase K (60 mg/mL), 5 µL of 1 M Tris-HCl, 1 µL of 0.5 M EDTA, 50 µL of 10% SDS, and 435.5 µL of sterile water. Samples were incubated at 65 °C for 24 h at 1200 rpm. DNA extraction was performed by adding 400 µL of chloroform and centrifugation at 12,000 rpm for 15 min. The supernatant was transferred to a 1.5 mL microcentrifuge tube, mixed with 50 µL of 5 M sodium acetate, and centrifuged again at 12,000 rpm for 15 min. Cold isopropanol was then added to precipitate the DNA, and the sample was purified with 70% ethanol. Finally, DNA was rehydrated in 60 µL of 10 mM Tris-HCl (pH 8.0) and 1 mM EDTA and stored at −20 °C until further use. DNA concentration was measured using a NanoDrop 2000 spectrophotometer (Thermo Scientific, Waltham, MA, USA), and quality was confirmed by electrophoresis in a 1% agarose gel.

### 2.4. PCR Amplification of the Miniexon Gene

For miniexon PCR, primers Fme (5′-TAT TGG TAT GCG AAA CTT CCG-3′) and Rme (5′-ACA GAA ACT GAT ACT TAT ATA GCG-3′) were used to amplify the nontranscribed spacer region of the miniexon sequence of *Leishmania* [17]. The PCR reactions were set up using the Phusion^®^ High-Fidelity DNA Polymerase kit (New England Biolabs, Ipswich, MA, USA) in a total volume of 25 µL. Each reaction contained 1X High Fidelity buffer, 1.25 µL of each primer at a final concentration of 0.5 µM, 3% dimethyl sulfoxide (DMSO), 0.5 µL of dNTPs (200 µM), 0.25 µL of Phusion DNA polymerase, 14 µL of nuclease-free water, and 2 µL of extracted DNA. Thermal cycling was performed using an Aeris™ PCR Thermal Cycler (ESCO Lifesciences Group, Singapore) under the following program: initial denaturation at 98 °C for 30 s, followed by 30 cycles of denaturation at 98 °C for 10 s, annealing at 59 °C for 30 s, and extension at 72 °C for 30 s. A final extension step was performed at 72 °C for 10 min, and the samples were subsequently held at 4 °C. PCR products were visualized by electrophoresis on a 2.5% agarose gel stained with SYBR™ Safe DNA Gel Stain (Invitrogen, Waltham, MA, USA). A volume of 10 µL of the PCR product was loaded per well. Amplification products were visualized by electrophoresis on a 2.5% agarose gel stained with 1 µL of SYBR™ Safe DNA Gel Stain (Invitrogen). Bands were photographed under UV illumination. Samples were considered positive if a band of the expected size was observed in at least two independent amplifications.

### 2.5. PCR Amplification of rRNA Gene Internal Transcribed Spacer 1 (ITS1)

To detect the internal transcribed spacer 1 (ITS1) region of the *Leishmania* genus, we employed the primers LITSR (forward): 5′-CTGGATCATTTTCCGATG-3′ and L5.8S (reverse): 5′-TGATACCACTTATCGCACTT-3′, as previously described by El Tai et al. [18] PCR reactions were prepared using the Phusion^®^ High-Fidelity (HF) DNA Polymerase kit (New England Biolabs, Ipswich, MA, USA) in a final reaction volume of 25 µL. The master mix contained 1X HF buffer, 1.25 µL of each primer at a final concentration of 0.5 µM, 3% DMSO, 0.5 µL of dNTPs (200 µM), 0.25 µL of Phusion polymerase, 14 µL of nuclease-free water, and 2 µL of template DNA. Amplification was performed in an Aeris™ PCR Thermal Cycler (ESCO Lifesciences Group, Singapore) under the following cycling conditions: an initial denaturation step at 98 °C for 30 s, followed by 30 cycles of denaturation at 98 °C for 10 s, annealing at 56 °C for 30 s, and extension at 72 °C for 30 s. A final extension was conducted at 72 °C for 10 min, and reactions were maintained at 4 °C.

To assess the presence of the expected ~350 bp amplicon, 10 µL of the PCR product was resolved on a 2.5% agarose gel stained with 1 µL of SYBR™ Safe DNA Gel Stain (Invitrogen, Carlsbad, CA, USA).

Positive controls included reference strains of *Leishmania* spp.: *L. donovani* (MHOM/SD/62/1S), *L. panamensis* (MHOM/PA/94/PSC-1), *L. tropica* (MHOM/AF/87/RUP), *L. major* (MHOM/SN/74/SD), *L. amazonensis* (provided by the Centro de Investigación de Enfermedades Tropicales—CINTROP). In addition, *Trypanosoma cruzi* DNA was included as a negative control to evaluate potential cross-reactivity.

### 2.6. RFLP Analysis of the ITS1 PCR Amplicon

PCR products (20 μL) were digested with HaeIII (New England Biolabs), following the manufacturer’s instructions. The restriction fragments were analyzed by electrophoresis at 120 V in 1× Tris-acetate-EDTA (TAE) buffer on 3% agarose gels. Fragments were visualized under ultraviolet light, and the sizes of the restriction products were determined. Genomic DNA reference sequences obtained from the NCBI GenBank database were used for the species *L. panamensis* (CP009396.1), *L. braziliensis* (MW538634.1), *L. amazonensis* (CM061637.1), *L. donovani* (FR799614.1), *L. major* (PQ157881.1), and *L. tropica* (CM024313.1). Subsequently, the binding sites of the LITSR and L5.8S primers were identified using SnapGene 8.1 software, which allowed for the determination of the expected amplicon size corresponding to the ITS1 region. Finally, enzymatic digestion with HaeIII was simulated using the Molbiotools Restriction Analyzer tool to compare the generated profiles with the experimental results and validate their concordance with the expected patterns.

To assess the quality and amplifiability of DNA extracted from FFPE tissue samples, the GAPDH housekeeping gene was amplified by real-time PCR in all samples included in the study. The GAPDH gene, a human-specific target, was chosen as an internal control to evaluate potential PCR inhibition. The primers utilized were GAPDH-F (5′-CCTAGGGCTGCTCACATATTC-3′) and GAPDH-R (5′-CGCCCAATACGACCAAATCTA-3′), which amplify an 80 bp fragment. Each PCR reaction comprised 5 µL of SYBR Green amplification buffer (Applied Biosystems, Hong Kong, China), 2.2 µL of ultrapure water, 0.5 µM of each primer, and 2 µL of genomic DNA at a concentration of 10 ng/µL, resulting in a final reaction volume of 10 µL. The amplification protocol consisted of an initial denaturation at 95 °C for 10 min, followed by 40 cycles of denaturation at 95 °C for 15 s and annealing and extension at 60 °C for 1 min. A melting curve analysis was then performed. Reactions were conducted using a StepOnePlus™ Real-Time PCR System (96-well format, Applied Biosystems). Each run included a negative control (no DNA template) and a positive control containing 10 ng/µL of human DNA.

### 2.7. Ethical Considerations

This study was conducted on archived biopsy samples collected during routine diagnostic procedures. All samples were anonymized. The research protocol was reviewed and approved by the Scientific Research Ethics Committee (CEINCI) of the Industrial University of Santander, under approval number (No. 12, 29 April 2024).

### 2.8. Statistical Analysis

A database was created using Microsoft Office 2024 tools to record sociodemographic variables, including the place of occurrence, sex, and age, along with clinical variables pertinent to the histopathological diagnosis of cutaneous leishmaniasis. These clinical variables encompassed the type of sample, anatomical site of the lesion, classification of cellular morphological patterns, and the extent of tissue involvement in the epidermis, dermis, and hypodermis. Additionally, results from molecular testing using PCR were incorporated. Cellular patterns were analyzed based on their presence or absence in each sample, and the degree of hypodermal involvement was categorized into three levels: mild, moderate, and extensive. Samples were considered true positives when *Leishmania* DNA was detected by at least one of the two PCR assays. Descriptive statistics, such as absolute and relative frequencies, were used to summarize the variables. Data were processed and analyzed using SPSS version 2.0, and the findings are presented in tables according to variable type.

## 3. Results

Sixteen patients were included in the present study, comprising 62.5% males and 37.5% females, with an age range of 4 to 60 years. The lower extremities were the most commonly affected sites (56.2%), followed by the upper extremities (37.5%), and the face. When examining the age distribution by life stage and sex, it was found that four of the ten male samples (40%) were from adults, while two samples (20%) were from children aged 0 to 5 years. Among the female population, one (17.7%) of the six (16.7%) were adolescents (Table 1).

### 3.1. Histopathological Findings

**Figure 1:**
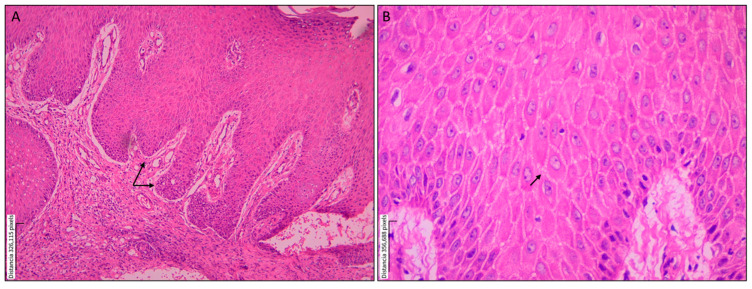
Epidermal alterations in a skin biopsy from a patient with localized cutaneous leishmaniasis. Paraffin-embedded histological section of a skin lesion from a patient with localized cutaneous leishmaniasis caused by *Leishmania* spp., stained with hematoxylin and eosin (H&E). (**A**) Acanthosis is characterized by irregular elongation of the epidermal rete ridges; magnification: 10×. (**B**) Spongiosis showing intercellular epidermal edema; magnification: 40×.

**Figure 2:**
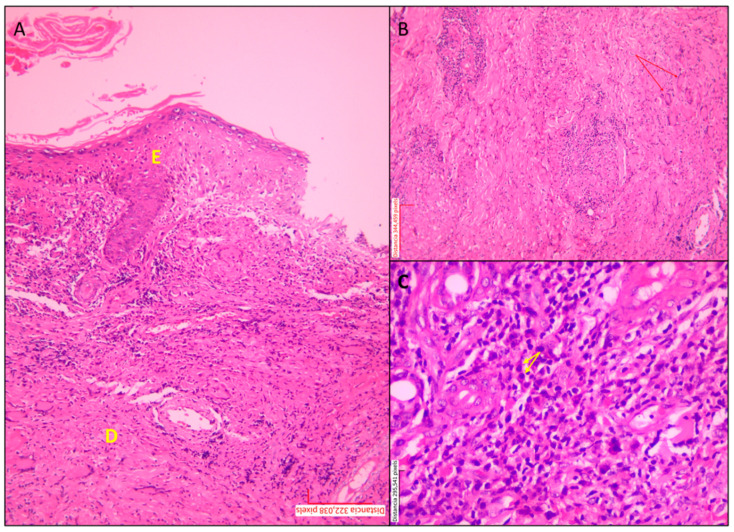
Histopathological inflammatory patterns in skin biopsies from patients with cutaneous leishmaniasis. Histopathological features of cutaneous leishmaniasis in skin biopsies stained with hematoxylin and eosin (H&E). (**A**) The panoramic view displays the epidermis (E) and the underlying dermis (D). Epidermal hyperplasia (E) with focal ulceration at the upper right margin. The underlying dermis (D) shows granuloma formation and a dense inflammatory infiltrate; magnification: 10×. (**B**) The deep dermis contains epithelioid granulomas, multinucleated giant cells (indicated by red arrows), and an abundance of lymphocytes and plasma cells; magnification: 20×. (**C**) Diffuse lymphoplasmacytic inflammatory pattern; yellow arrows highlight plasma cells; magnification: 40×.

**Figure 3:**
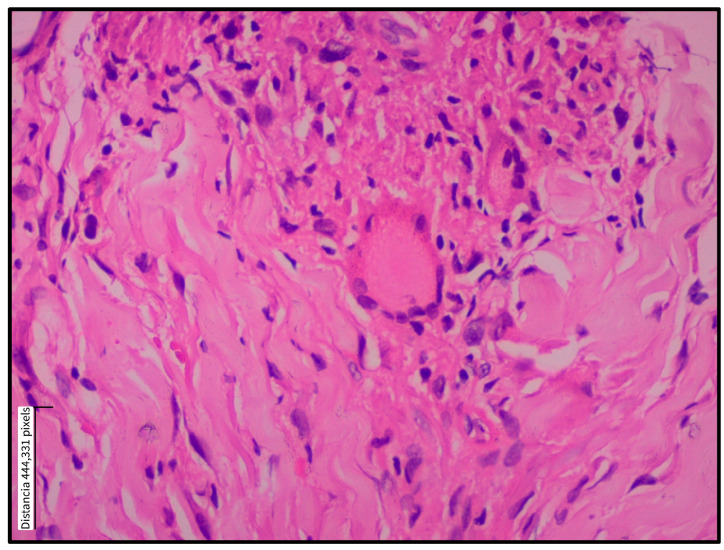
Multinucleated giant cell within a dermal granuloma in cutaneous leishmaniasis. Multinucleated giant cell in the dermis of a skin biopsy from a patient with localized cutaneous leishmaniasis. A paraffin-embedded histological section stained with hematoxylin and eosin shows a multinucleated giant cell (center) surrounded by an inflammatory infiltrate composed predominantly of lymphocytes and histiocytes. Collagen bundles appear displaced by the cellular infiltrate. Magnification: 40×.

Histopathological findings were classified into epidermal and dermal alterations, as summarized in Table 2. The most frequently observed epidermal changes were acanthosis (93.8%), pseudoepitheliomatous hyperplasia (87.5%), and spongiosis (81.3%)—Figure 1. In two patients, evaluation of the epidermis was not possible because of ulceration and insufficient epidermal tissue. Most patients had biopsies showing a diffuse granulomatous inflammatory pattern, while only two patients exhibited a nodular pattern. Regarding dermal alterations, the inflammatory infiltrate consisted of granulomatous structures 12 (75.1%), accompanied by a mixed population of inflammatory cells, predominantly plasma cells, lymphocytes, and histiocytes. A marked abundance of these cells was observed in 11 (68.8%), 13 (81.3%), and 6 (37.5%) cases, respectively. Epithelioid granuloma was the most commonly observed type (56.3%), followed by tuberculoid (18.8%), as shown in Figure 2. Multinucleated Langhans-type giant cells were identified in the inner part of the granuloma (37.5%), as shown in Figure 3. All sections were re-examined for the presence of amastigotes, which were found in only one specimen.

### 3.2. PCR Detection of Leishmania DNA

**Figure 4:**
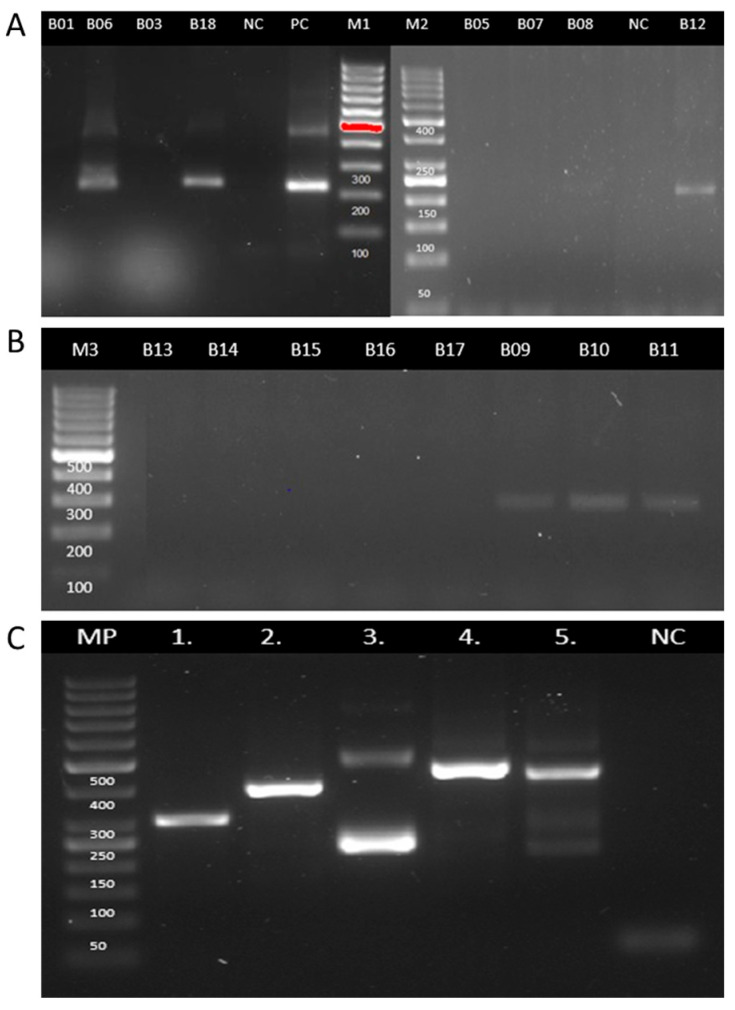
Electrophoretic analysis of PCR products from miniexon genes in FFPE tissue samples for detecting *Leishmania*. Electrophoretic separation (2.5% agarose gel) of Leishmania-specific polymerase chain reaction (PCR) miniexon gene fragments (*L. Leishmania*: 283–327 bp—*L. Viannia*: 223–226 bp). (**A**) Lanes B01, B03, B05, B06, B07, B08, B18, B12: Samples of formalin-fixed paraffin-embedded tissue (FFPE). NC: Negative Control. PC: Positive control, *L. (V) panamensis* NR 50,162 (MHOM/Pa/94/psc-1). M1: DNA Marker Gen Ruler 100 bp. M2: DNA Marker Gen Ruler 50 bp. (**B**) Lanes B09, B10, B11, B13, B14, B15, B16, B17: Samples of formalin-fixed paraffin-embedded tissue (FFPE). M3: DNA Marker Gen Ruler 100 bp. (**C**) Polymerase chain reaction–miniexon gene amplification products from different *Leishmania* reference strains lane 1, L *L. (L) amazonensis* strains CINTROP; lane 2, *L. (L) donovani* 915 (MHOM/IN 95/9515); lane 3, *L. (V) panamensis* NR 50,162 (MHOM/Pa/94/psc-1); lane 4, *L. (L) major* NIHSD (MHOM/SN/74/SD); lane 5, *L. (L) tropica* NIH RPU (MHOM/AF/87/RUP); lane 6, negative control; MP: 50 bp molecular size marker.

**Figure 5:**
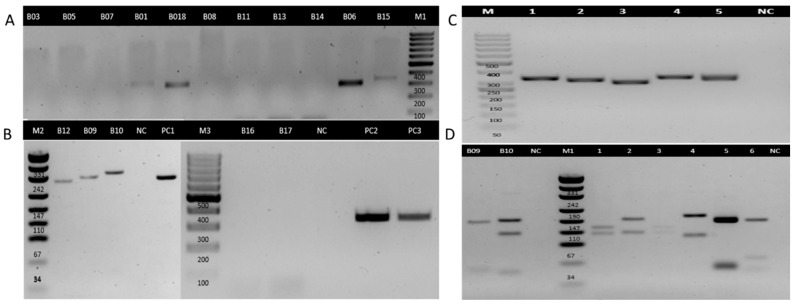
Electrophoretic analysis of PCR products from the ITS1 genes in FFPE tissue samples for detecting *Leishmania*. Electrophoretic separation (2, 5% agarose gel) of Leishmania-specific polymerase chain reaction (PCR) ITS1 region fragments (300–400 bp). (**A**) Lanes B01, B03, B05, B06, B07, B08, B11, B13, B14, B15, B18: Samples of formalin-fixed paraffin-embedded tissue (FFPE). M1: DNA Marker Gen Ruler 100 bp. (**B**) Lanes B09, B10, B12, B16, B17: Samples of formalin-fixed paraffin-embedded tissue (FFPE). M2: DNA Marker puc19. NC: Negative Control. PC1: Positive control, *L. (V) panamensis* NR 50,162 (MHOM/Pa/94/psc-1). M3: DNA Marker Gen Ruler 100 bp. PC2: Positive control, *L. (L) major* NIHSD (MHOM/SN/74/SD). PC3: Positive control, *L. (L) tropica* NIH RPU (MHOM/AF/87/RUP) (225 and 100 bp). (**C**) Polymerase chain reaction–ITS1 region amplification products from different *Leishmania* reference strains and clinical samples: lane 1, L *L. (L) amazonensis* strains CINTROP; lane 2, *L. (L) donovani* 915 (MHOM/IN 95/9515); lane 3, *L. (V) panamensis* NR 50,162 (MHOM/Pa/94/psc-1); lane 4, *L. (L) major* NIHSD (MHOM/SN/74/SD); lane 5, *L. (L) tropica* NIH RPU (MHOM/AF/87/RUP); lane NC Negative control; MP: 50 bp molecular size marker. (**D**) Agarose gel electrophoresis (3%) of the PCR-RFLP method using the Hae III restriction enzyme. (M1) puc19 DNA ladder. B09, B10: Samples of formalin-fixed paraffin-embedded tissue (FFPE). Lane 1 to 6 was positive to (1) *L. (V) panamensis* NR 50,162 (MHOM/Pa/94/psc-1) (150 and 147 bp) bands; (2) L *L. (L) amazonensis* strains CINTROP (210 and 147 bp); (3) *Leishmania braziliensis* (150 and 147 bp); (4) *L. (L) major* NIHSD (MHOM/SN/74/SD) (206 and 132 bp); (5) *L. (L) tropica* NIH RPU (MHOM/AF/87/RUP) (225 and 100 bp)—NC: negative control.

No amplification was observed in the negative controls, while all positive controls yielded the expected results. Additionally, all clinical samples produced amplification products of the expected size for the GAPDH housekeeping gene, confirming that the extracted DNA was of sufficient quality for downstream molecular analysis. Miniexon-PCR enabled the detection of *Leishmania* DNA in six samples (Figure 4, Table 3). Based on the size of the amplicons, subgenus-level identification was achieved in all positive cases: three samples exhibited a banding pattern consistent with the *Viannia* subgenus, and the other three with the *Leishmania (Leishmania)* subgenus (Table 3). In parallel, ITS1-PCR was positive in seven samples (43.8%) (Figure 5, Table 3). RFLP analysis of the ITS1-PCR products was carried out using the HaeIII restriction enzyme to determine the species or subgenus of *Leishmania* present in the samples. Of the seven samples that tested positive by ITS1-PCR, only two yielded interpretable digestion patterns. One sample showed a restriction profile compatible with the *Leishmania (Leishmania)*, which allowed the typification until the subgenus level. The other sample exhibited a pattern consistent with *Leishmania (L.) amazonensis*. The remaining five samples did not produce discernible digestion profiles, possibly due to low DNA concentration, partial digestion, or suboptimal band resolution.

Five cases were positive for both targets, and three were positive for only one of the two assays Table 3.

The detection limit of the PCR assay was evaluated using serial dilutions of *L. amazonensis* (miniexon gene) and *L. panamensis* (ITS1 region) DNA, ranging from 100 to 0.0001 ng/μL. The minimum DNA concentration that produced visible amplification was 0.001 ng/μL, establishing the assay’s analytical sensitivity under the experimental conditions.

## 4. Discussion

In our research, we employed two molecular targets—ITS1 and miniexon—for the detection of *Leishmania* DNA in FFPE skin biopsy samples from patients suspected of having CL. PCR amplification of the ITS1 region produced positive results in 7 (43.8%) out of 16 samples, while the miniexon target was positive in 6 (37.5%) cases. The use of both molecular targets resulted in a 50% detection rate, thereby improving the overall diagnostic performance. This dual-target approach may be particularly valuable for samples with low parasite loads or degraded DNA.

Our findings are consistent with those of Laskay et al., 1995, who detected *Leishmania* DNA in 7 out of 22 microscopically negative cases (31.8%) using a kinetoplast DNA target [19]. Similarly, Momeni et al. reported a 48% detection rate (30/63) in chronic lupoid leishmaniasis [20]. Weigle et al. [21] analyzed FFPE samples from patients in Cali, Colombia. They observed a higher sensitivity in acute lesions (75.5%) compared to chronic cases (45.5%), highlighting the reduced performance of PCR in long-standing lesions [21]. This trend aligns with our results, as most of the samples analyzed in our study corresponded to chronic or late-stage lesions, in which a lower parasite burden is typically expected.

In our study, *Leishmania* detection using PCR targeting the ITS1 region yielded a sensitivity of 43.8% in FFPE skin samples with suspected CL. This sensitivity is lower than that reported in other studies, such as those by Safaei et al. (2002) and Lanús et al. (2005), who used kinetoplast DNA (kDNA) as the molecular target, achieving sensitivities of 61% and 92%, respectively, in similar FFPE samples [22,23]. This difference may be partly due to the high copy number of kDNA compared to ribosomal targets, which favors greater analytical sensitivity. However, in our study, ITS1 showed a slightly higher positivity rate, which could reflect a marginally better performance in FFPE tissues, possibly related to its moderate amplicon size [24]. Although detection using miniexon yielded positive results in only 6 out of 16 samples, this marker remains useful as a confirmatory tool due to its high conservation among *Leishmania* species. The low positivity rate observed in our study may be influenced by the limited quantity and quality of *Leishmania* DNA in FFPE tissues. Similar findings were reported by Dietrich et al. (2013), who also evaluated miniexon in paraffin-embedded skin samples and attributed the low performance not only to extensive DNA degradation but also to the potential inhibitory effect of small, fragmented DNA on polymerase activity—an issue commonly encountered with FFPE-derived nucleic acids [25].

The use of both targets in parallel increases the likelihood of detecting parasite DNA in challenging clinical specimens, reinforcing the role of multiplexed molecular strategies in improving diagnostic accuracy [25]. While this result highlights the potential of PCR as a confirmatory tool, the sensitivity observed here should be interpreted with caution, given the small sample size and the lack of a universally accepted reference standard for confirming the diagnosis in all cases. Our findings support the relevance of implementing molecular diagnostics as a complementary tool in endemic areas, such as Colombia, where conventional methods may yield inconclusive results. The use of PCR in FFPE samples could enhance the diagnostic capacity in routine pathology laboratories without requiring fresh tissue, making it feasible and scalable in low-resource settings [26]. Given Colombia’s persistent incidence of CL, especially in departments such as Santander, integrating PCR into routine diagnostics could contribute to earlier confirmation, improved case management, and better surveillance strategies.

Molecular characterization of the seven samples positive for *Leishmania* DNA allowed partial taxonomic classification at the genus or subgenus level. Three samples were identified as belonging to the *Viannia* subgenus, two to the *Leishmania* subgenus, and two could only be assigned at the genus level (*Leishmania* spp.). These findings reflect the diversity of *Leishmania* species circulating in endemic regions of Colombia and are consistent with previous reports describing the geographic distribution of *L. panamensis*, *L. braziliensis*, and, to a lesser extent, *L. amazonensis* in departments such as Antioquia and Meta [27]. Although species-level identification was not achieved in all cases, the subgenus-level classification provides valuable epidemiological insight, particularly in endemic settings where multiple *Leishmania* species coexist [28].

On the other hand, eight samples did not yield detectable amplification despite presenting granulomatous inflammatory patterns suggestive of CL. Several factors could explain these negative results. First, it is possible that these lesions were caused by other granulomatous dermatoses that mimic leishmaniasis both clinically and histologically, such as deep fungal infections, atypical mycobacterioses, sarcoidosis, or foreign body granulomas [29]. Second, technical limitations inherent to FFPE tissue may have contributed, including DNA degradation, low parasite burden, or the presence of PCR inhibitors [30]. The inclusion of two FFPE blocks with confirmed amastigotes as positive controls demonstrated successful amplification under the conditions used, supporting the adequacy of the protocol. Additionally, two FFPE skin biopsies from non-leishmaniasis dermatological conditions were used as negative controls, and no amplification was observed, suggesting the absence of nonspecific amplification or contamination. Therefore, while PCR improves detection in histopathologically ambiguous cases, its sensitivity may still be affected by intrinsic characteristics of the sample or by the biology of the lesion itself [31].

This study has some limitations that should be acknowledged. First, the sample size was relatively small (*n* = 16), which limits the generalizability of the findings and precludes robust statistical analysis of diagnostic accuracy parameters, such as sensitivity, specificity, and predictive values. Nonetheless, the results provide preliminary yet meaningful evidence supporting the utility of PCR for confirming CL in skin biopsies with inconclusive histopathology. Additionally, it is essential to note the increased cost associated with using two PCR protocols, which may pose a constraint in resource-limited settings.

Second, the absence of an additional gold standard, such as parasite culture, immunohistochemistry, or follow-up clinical response, may have limited the ability to independently verify the PCR findings [32]. However, all cases included in this study had clinical and histological features strongly suggestive of leishmaniasis, thereby strengthening the relevance of the molecular results.

Finally, as this was a retrospective analysis of FFPE tissue, DNA degradation or cross-contamination may have influenced PCR performance. Future studies with larger, prospectively collected samples and multimodal diagnostic confirmation are needed to validate and expand upon these findings.

In conclusion, our results suggest that integrating molecular diagnostics with histopathological evaluation may improve the diagnostic accuracy of CL, particularly in cases with inconclusive histopathological findings. While preliminary, these findings support the potential value of a combined approach, especially for chronic lesions where conventional methods are limited. This strategy could complement existing diagnostic workflows, particularly in endemic regions.

## Figures and Tables

**Table 1:** Demographic and clinical characteristics of the study population.

Characteristic	Category	*n* = 16 (%)
Sex	Male	10 (62.5%)
	Female	6 (37.5%)
Age group by sex		
Male (*n* = 10)	Children (0–5 yrs)	2 (20.0%)
	Adolescents (10–17 yrs)	1 (10.0%)
	Adults (18–59 yrs)	4 (40.0%)
	Older adults (60+)	3 (30.0%)
Female (*n* = 6)	Children (0–5 yrs)	1 (16.7%)
	Adolescents (10–17 yrs)	1 (16.7%)
	Adults (18–59 yrs)	3 (50.0%)
	Older adults (60+)	1 (16.7%)
Site of lesion	Lower extremities	9 (56.2%)
	Upper extremities	6 (37.5%)
	Head and face	1 (6.3%)

The table summarizes the distribution by sex, age group, and site of lesion. Percentages are based on the total number of patients in each subgroup.

**Table 2:** Epidermal and dermal histopathological changes in skin biopsies from patients with suspected cutaneous leishmaniasis.

Tissue Level	Histological Feature	*n* = 16 (%)	
Epidermis	Ulceration	14 (87.5)	
	Acanthosis	15 (93.8)	
	Pseudoepitheliomatous hyperplasia	14 (87.5)	
	Spongiosis	13 (81.3)	
	Parakeratosis	13 (81.3)	
	Transepidermal migration	8 (50.0)	
Dermis	Inflammation	15 (93.8)	
	Granulomatous pattern	10 (62.5)	
	Fibrosis	10 (62.5)	
	Vascular proliferation	13 (81.3)	
Vascular type	Telangiectasias	6 (37.5)	
	Prominent endothelium	8 (50.0)	
Inflammatory cell type		Abundant	Scant
	Plasma cells	11 (68.8)	5 (31.3)
	Lymphocytes	13 (81.3)	3 (18.8)
	Histiocytes	6 (37.5)	10 (62.5)
	Infiltrate classification	Mixed-type granuloma (100)	
Infiltrate distribution	Perivascular	0 (0)	
	Interstitial	0 (0)	
	Diffuse	14 (87.5)	
	Nodular	2 (12.5)	
Granulomas	Tuberculoid	3 (18.8)	
	Epithelioid	9 (56.3)	
	Sarcoid	0 (0)	
Giant cells	Multinucleated	3 (18.8)	
	Langhans-type multinucleated	6 (37.5)	

The table summarizes key epidermal and dermal alterations, including inflammatory and vascular changes, distribution and type of inflammatory infiltrates, granuloma subtype, and presence of giant cells. Frequencies are expressed as the number of cases and percentages. “Abundant” and “scant” refer to the relative quantity of inflammatory cell types observed in the infiltrate.

**Table 3:** Summary of histopathological interpretation and molecular detection of *Leishmania* DNA in FFPE skin biopsy samples.

		Histopathological Findings	Molecular Testing				
Patient	Date of Sample Collection	Case Definition	Miniexon		ITS1		Molecular Characterization
			PCR	Subgenus *	PCR	RFLP **	
B01	2024	Suggestive	Negative	NA	Positive	NA	*Leishmania* spp.
B03	2021	Suggestive	Negative	NA	Negative	NA	NA
B05	2021	Suggestive	Negative	NA	Negative	NA	NA
B06	2023	Suggestive	Positive	*L. Viannia*	Positive	NA	*L. Viannia*
B07	2021	Suggestive	Negative	NA	Negative	NA	NA
B08	2021	Suggestive	Negative	NA	Negative	NA	NA
B09	2021	Suggestive	Positive	*L. Leishmania*	Positive	*L. Leishmania*	*L. Leishmania*
B10	2023	Suggestive	Positive	*L. Leishmania*	Positive	*L. Leishmania*	*L. Leishmania*
B11	2023	Suggestive	Positive	*L. Leishmania*	Negative	NA	*L. Leishmania*
B12	2024	Suggestive	Positive	*L. Viannia*	Positive	NA	*L. Viannia*
B13	2021	Suggestive	Negative	NA	Negative	NA	NA
B14	2021	Suggestive	Negative	NA	Negative	NA	NA
B15	2024	Suggestive	Negative	NA	Positive	NA	*Leishmania* spp.
B16	2024	Suggestive	Negative	NA	Negative	NA	NA
B17	2024	Suggestive	Negative	NA	Negative	NA	NA
B18	2024	Suggestive	Positive	*L. Viannia*	Positive	NA	*L. Viannia*

Suggestive: The biopsy shows a dense inflammatory infiltrate without evidence of amastigotes. NA: Test not applied. Subgenus *: The identification of the subgenus is based on the visualization of the amplicons: *Leishmania (Leishmania)* produces bands of 283–327 bp, while *Leishmania (Viannia)* produces bands of 223 bp. RFLP **: Subgenus or species can be identified by observing the amplicon profiles after digestion with the HaeII enzyme.

## Data Availability

Study data are available from the corresponding author upon request.

## References

[1] Arenas R., Torres-Guerrero E., Quintanilla-Cedillo M.R., Ruiz-Esmenjaud J. (2017). Leishmaniasis: A review. F1000Research.

[2] OMS (2023). Leishmaniasis—PAHO/WHO|Pan American Health Organization. Pan American Health Organization. https://www.who.int/news-room/fact-sheets/detail/leishmaniasis.

[3] Pan American Health Organization (2019). Manual of Procedures for Surveillance and Control of Leishmaniasis in the Americas. World Health Organization. https://iris.paho.org/handle/10665.2/51838.

[4] Bennis I., De Brouwere V., Belrhiti Z., Sahibi H., Boelaert M. (2018). Psychosocial burden of localised cutaneous Leishmaniasis: A scoping review. BMC Public Health.

[5] Bilgic-Temel A., Murrell D.F., Uzun S. (2019). Cutaneous leishmaniasis: A neglected disfiguring disease for women. Int. J. Women’s Dermatol..

[6] Bailey M.S., Lockwood D.N.J. (2007). Cutaneous Leishmaniasis. Clin. Dermatol..

[7] Komurcugil I., Nek K.N. (2022). Cutaneous Leishmaniasis with Unusual Psoriasiform Presentation. J. Turk. Acad. Dermatol..

[8] Guimarães L.H., Queiroz A., Silva J.A., Silva S.C., Magalhães V., Lago E.L., Machado P.R.L., Bacellar O., Wilson M.E., Beverley S.M. (2016). Atypical Manifestations of Cutaneous Leishmaniasis in a Region Endemic for *Leishmania braziliensis*: Clinical, Immunological and Parasitological Aspects. PLoS Negl. Trop. Dis..

[9] Meireles C.B., Maia L.C., Soares G.C., Teodoro I.P.P., Gadelha M.D.S.V., da Silva C.G.L., de Lima M.A.P. (2017). Atypical presentations of cutaneous leishmaniasis: A systematic review. Acta Trop..

[10] Piyasiri S.B., Dewasurendra R., Samaranayake N., Karunaweera N. (2023). Diagnostic Tools for Cutaneous Leishmaniasis Caused by *Leishmania donovani*: A Narrative Review. Diagnostics.

[11] Reina A.M., Mewa J.C., Calzada J.E., Saldaña A. (2022). Characterization of *Leishmania* spp. Causing Cutaneous Lesions with a Negative Parasitological Diagnosis in Panama. Trop. Med. Infect. Dis..

[12] Wijesinghe H.D., Wijesinghe G.K., Fernando D., de Silva C. (2022). Immunopathology of Cutaneous Leishmaniasis in a Cohort of Sri Lankan Patients. Clin. Pathol..

[13] de Vries H.J.C., Schallig H.D. (2022). Cutaneous Leishmaniasis: A 2022 Updated Narrative Review into Diagnosis and Management Developments. Am. J. Clin. Dermatol..

[14] Mesa L.E., Manrique R., Muskus C., Robledo S.M. (2020). Test accuracy of polymerase chain reaction methods against conventional diagnostic techniques for cutaneous leishmaniasis (Cl) in patients with clinical or epidemiological suspicion of cl: Systematic review and meta-analysis. PLoS Negl. Trop. Dis..

[15] Greytak S.R., Engel K.B., Bass B.P., Moore H.M. (2015). Accuracy of molecular data generated with FFPE Biospecimens: Lessons from the literature. Cancer Res..

[16] Casaril A.E., de Oliveira L.P., Alonso D.P., de Oliveira E.F., Barrios S.P.G., Infran J.d.O.M., Fernandes W.d.S., Oshiro E.T., Ferreira A.M.T., Ribolla P.E.M. (2017). Standardization of DNA extraction from sand flies: Application to genotyping by next generation sequencing. Exp. Parasitol..

[17] Marfurt J., Nasereddin A., Niederwieser I., Jaffe C.L., Beck H.P., Felger I. (2003). Identification and differentiation of *Leishmania* species in clinical samples by PCR amplification of the miniexon sequence and subsequent restriction fragment length polymorphism analysis. J. Clin. Microbiol..

[18] El Tai N.O., El Fari M., Mauricio I., Miles M.A., Oskam L., El Safi S.H., Presber W.H., Schönian G. (2001). *Leishmania donovani*: Intraspecific polymorphisms of Sudanese isolates revealed by PCR-based analyses and DNA sequencing. Exp. Parasitol..

[19] Laskay T., Mikó T.L., Negesse Y., Solbach W., Röllinghoff M., Frommel D. (1995). Detection of cutaneous *leishmania* infection in paraffin-embedded skin biopsies using the polymerase chain reaction. Trans. R. Soc. Trop. Med. Hyg..

[20] Momeni A.Z., Yotsumoto S., Mehregan D.R., Mehregan A.H., Mehregan D.A., Aminjavaheri M., Fujiwara H., Tada J. (1996). Chronic lupoid leishmaniasis: Evaluation by polymerase chain reaction. Arch. Dermatol..

[21] Weigle K.A., Labrada L.A., Lozano C., Santrich C., Barker D.C. (2002). PCR-based diagnosis of acute and chronic cutaneous leishmaniasis caused by *Leishmania* (Viannia). J. Clin. Microbiol..

[22] Safaei A., Motazedian M.H., Vasei M. (2002). Polymerase chain reaction for diagnosis of cutaneous leishmaniasis in histologically positive, suspicious and negative skin biopsies. Dermatology.

[23] Lanús E.C., Piñero J.E., González A.C., Valladares B., De Grosso M.L., Salomón O.D. (2005). Detection of *Leishmania braziliensis* in human paraffin-embedded tissues from Tucumán, Argentina by polymerase chain reaction. Memórias Do Inst. Oswaldo Cruz.

[24] Toz S.O., Culha G., Zeyrek F.Y., Ertabaklar H., Alkan M.Z., Vardarlı A.T., Gunduz C., Ozbel Y., Louzir H. (2013). A Real-Time ITS1-PCR Based Method in the Diagnosis and Species Identification of *Leishmania* Parasite from Human and Dog Clinical Samples in Turkey. PLoS Negl. Trop. Dis..

[25] Dietrich D., Uhl B., Sailer V., Holmes E.E., Jung M., Meller S., Kristiansen G., Castresana J.S. (2013). Improved PCR Performance Using Template DNA from Formalin-Fixed and Paraffin-Embedded Tissues by Overcoming PCR Inhibition. PLoS ONE.

[26] Gebhardt M., Ertas B., Falk T.M., Blödorn-Schlicht N., Metze D., Böer-Auer A. (2015). Fast, sensitive and specific diagnosis of infections with *Leishmania* spp. in formalin-fixed, paraffin-embedded skin biopsies by cytochrome b polymerase chain reaction. Br. J. Dermatol..

[27] Yuil J.M.R., Saldaña A., Calzada J., Arias J., Díaz R., González K. (2012). Comparación entre histopatología y PCR, para diagnóstico de leishmaniasis tegumentaria. Dermatol. Cosmet. Medica Y Quir..

[28] van der Auwera G., Dujardina J.C. (2015). Species typing in dermal leishmaniasis. Clin. Microbiol. Rev..

[29] Chatterjee D., Bhattacharjee R., Saikia U.N. (2021). Non-infectious granulomatous dermatoses: A pathologist’s perspective. Indian Dermatol. Online J..

[30] Babouee Flury B., Weisser M., Prince S.S., Bubendorf L., Battegay M., Frei R., Goldenberger D. (2014). Performances of two different panfungal PCRs to detect mould DNA in formalin-fixed paraffin-embedded tissue: What are the limiting factors?. BMC Infect. Dis..

[31] Braun-Falco M., Schempp W., Weyers W. (2008). Molecular diagnosis in dermatopathology: What makes sense, and what doesn’t. Exp. Dermatol..

[32] Thakur S., Joshi J., Kaur S. (2020). Leishmaniasis diagnosis: An update on the use of parasitological, immunological and molecular methods. J. Parasit. Dis..

